# Multi-Objective Optimization of Motor Sealing Performance: Numerical and Experimental Approach

**DOI:** 10.3390/ma18092064

**Published:** 2025-04-30

**Authors:** Weiru Zhou, Zonghong Xie

**Affiliations:** School of Aeronautics and Astronautics, Sun Yat-Sen University, Shenzhen 518107, China; zhouwr5@mail2.sysu.edu.cn

**Keywords:** rubber seal, numerical model, waterproof, multi-objective optimization

## Abstract

Rubber seals have been widely applied in mechanical sealing structures in various fields such as automobiles, aerospace, and deep-sea hydraulic systems. The current analysis methods for O-ring sealing performance mainly include experiments and simulations. This study takes the motor sealing structure as the research object and proposes a multi-objective optimization method for designing sealing structures. Based on the finite element analysis model, the main indicators related to sealing performance were obtained. These indicators transfer to multi-objective optimization analysis to determine the influence of different groove depths on sealing performance. The analysis results show that when the bolt preload is 50 N, a groove depth of 0.9 mm is the optimal design scheme. The optimal relationship between the O-ring diameter D and the sealing structure groove depth is H = 0.6 D. Moreover, a prototype test under the condition of IPX7 requirement verifies the optimal design scheme’s waterproof performance. The proposed method provides multiple design schemes for comprehensive evaluation considering different sealing structures. It reveals that the sealing performance is not only determined by rubber material characteristics but also by seal structure dimension.

## 1. Introduction

Rubber is widely used in sealing structures in various fields such as automobiles [1], aerospace [2], mechanical equipment [3,4,5], aviation industry [6,7], nuclear power plants [8], and deep-sea hydraulic systems [9,10]. A rubber seal tightly conforms to the interface through elastic deformation to prevent the leakage of liquids and gases. Its advantages include high temperature resistance, corrosion resistance, and aging resistance, as well as low cost and convenient installation [11].

**Figure 1:**
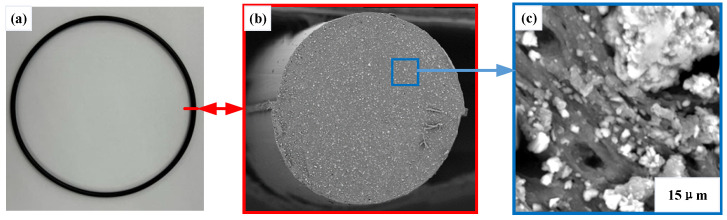
O-ring and its microstructure: (**a**) O-ring macrostructure, (**b**) section plane of O-ring, and (**c**) microstructure of O-ring.

The seals are robust, leakage-proof, and able to withstand harsh media and temperature, and endure performance and maintenance conditions. Common static face sealing options include flat gaskets, caulks, cements, RTVs, and O-rings. The O-ring and its microstructure are shown in Figure 1. Its surface is smooth while its interior is full of holes.

Previous studies have systematically analyzed O-ring sealing performance through simulation methods and experimental tests. A.F. George, A. Strozzi, and J.I. Rich wrote a finite element stress analysis computer program named FEMALES which is specifically for the analysis of large deformations in elastomeric materials [12]. Contact stress and area were studied by Shukla and Nigam through a general method from full-field photoelastic data [13]. A theoretical framework was established by Eugenio Dragoni and Antonio Strozzi to characterize the mechanical response of elastomeric O-ring seals under unpressurized conditions when seated in rectangular cross-section grooves [14]. For the nonlinear finite element analysis of axisymmetric rubber, Isaac Fried and Arthur R. Johnson gave a detailed derivation of the element gradient vector and stiffness matrix [15]. Kazutaka Yokoyama, Masayuki Okazaki, and Taku Komito analyzed the sealing ability of the O-ring and found that a large compression ratio is good for reliability [16]. To study more parameters that influence O-ring such as contact force, contact pressure, and geometry, Karaszkiewicz constructed formulas that analyze an O-ring that is mounted in a seal groove and exists in a condition of being loaded or unloaded with the sealed pressure [17]. For the von Mises stress and contact pressure, O-ring at different compression ratios and oil pressures were considered by Zhou and Zhang [18]. Morrell, P. R., M. Patel, and A. R. Skinner indicate that through experimental methods when rubber is under the condition of oxidative cross-linking, it will become hard and brittle [19]. Hyung-Kyu Kim et al. indicate that Lindley’s formulae show similar results to the finite element analysis results, but are lower than the measured contact force [20]. By using the ANSYS finite element analysis software for numerical simulation, the influence of the O-ring on the end-face deformation of the flexible ring of the mechanical seal was analyzed. The analysis results indicate that the end-face deformation of the flexible ring is directly affected by the compression ratio of the O-ring. With further analysis, another conclusion was reached: as the compression ratio increases, the radial taper of the sealing surface increases [21]. For O-rings exposed to high-pressure hydrogen cycles, the associations among hydrogen pressure, ambient temperature, pressure cycle mode and fracture characteristics were studied by Junichiro Yamabe et al. [22]. To study the failure causes of the O-ring of the water hydraulic pump, Zhibin Zhang et al. analyzed the two-dimensional finite element model of the rubber material and found that the main failure cause was the swing clearance between the sealing ring and the piston bushing [23]. Christopher Porter et al. conducted a large number of experimental studies on O-rings for aerospace sealing applications [24]. The research results show that the elongation at break of O-rings decreases with the increase in aging time, while the micro hardness, tensile strength, and 50% tensile stress show the opposite trend. For the shield machine sealing problem, Mei Yang et al. established a three-dimensional finite element analysis model to analyze the contact stress of O-rings with skeletons when the gate is opened and closed [25].

The focus of the prior studies was on the sealing performance of rubber rather than conducting optimization analysis on the sealing structure. Therefore, in this study, a three-dimensional finite element model of the motor sealing structure was established. There are three contact pairs around O-rings and the contact type is ‘frictional’. Under this setting, before relative sliding occurs, the two contact surfaces can transfer a certain amount of shear stress through the contact area. It is a type of nonlinear contact mode that best conforms to engineering practice [26].

The E-TOPSIS method, as a comprehensive evaluation method, has a wide range of applications in the field of engineering [27,28,29,30,31,32]. For the sealing structure optimization study, the best fit of groove depth and O-ring diameter using the entropy method and TOPSIS method is required. O-ring contact pressure and area are PIS parameters while O-ring stress and base stress are NIS parameters. By these parameters and the TOPSIS method, the best sealing structure is carried out. Furthermore, the sealing reliability of the sealing structure underwater tests the reliable sealing demand for waterproof quality. This study provides a new perspective for predicting the seal performance.

## 2. Models

### 2.1. Physical Model

**Figure 2:**
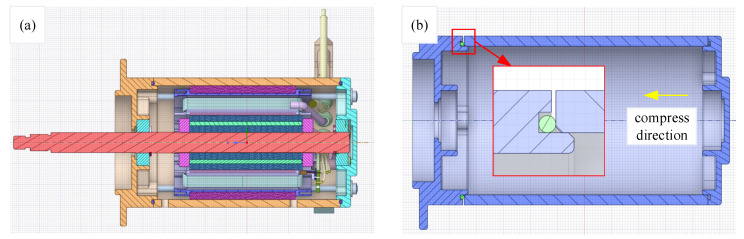
Section of motor model: (**a**) motor component parts; (**b**) simplified model.

Figure 2a shows the motor inside without simplification. The O-ring is installed in the groove, and when the motor shell compresses the O-ring in an axial direction under the pretension of the bolt, the O-ring will compress to the groove. To save computing time and computing resources, the model was simplified into 4 parts, which are the base, O-ring, shell, and cover in Figure 2b. The compression direction is also shown in Figure 2b. Due to its simple structure and low cost, nitrile butadiene rubber (NBR) is widely used in sealing structures [33]. Therefore, NBR material was selected in this study.

### 2.2. Numerical Model

The numerical method is often used to predict mechanical behavior in many fields of research [3,34,35,36,37]. The mechanical properties are predicted by computer simulation and it saves the consumption of labor and experimental consumables. Rubber’s mechanical behavior involves geometric nonlinearity and contact nonlinearity from surface interactions and friction during loading [18]. In this research, the subsequent assumptions were posited: the rubber seal remains unaffected by the medium temperature and creep, and does not influence the volume of the O-ring. Additionally, the consideration of all the materials being isotropic and homogeneous was incorporated. As rubber’s Poisson ratio is close to 0.5, its mechanical property is incompressible [38]. The Mooney–Rivlin two-parameter model was employed to simulate the performance of sealing, as this model is capable of describing the hyperelastic characteristics [39,40]. ψ is the strain energy density function [41]:

(1) $$\psi =C_{10\;}\left(\bar{I_{1}}-3\right)+C_{01\;}\left(\bar{I_{2}}-3\right)+\frac{1}{d}\;{\left(J-1\right)}^{2}$$

$$C_{10}=0.21\;MPa;\;C_{01}=0.053\;MPa;\;d=0.0012\;MPa$$

where *J* is the Jacobian matrix, *C*_10_ and *C*_01_ are the material constants, and *d* is the material incompressibility parameter (ANSYS Inc., Canonsburg, PA, USA, 2023).

**Figure 3:**
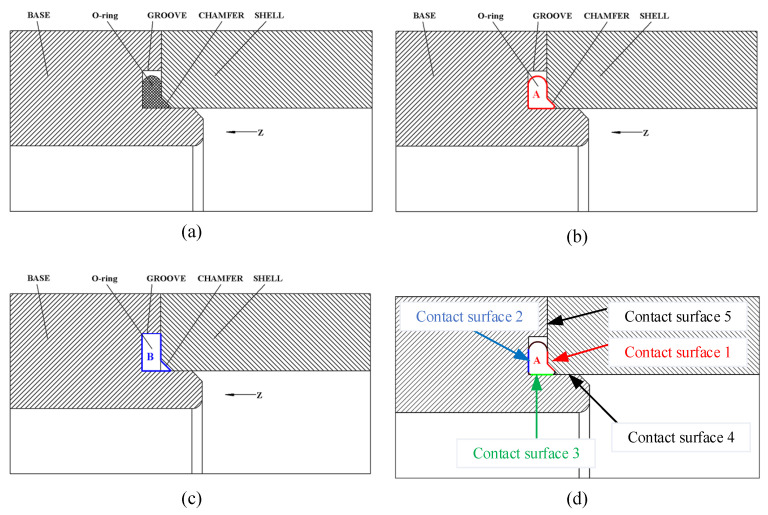
Sealing structure (**a**) Sealing structure cross-section, (**b**) O-ring compressed cross-section, (**c**) groove cross-section, (**d**) O-ring contact surfaces.

The outline drawing of the seal component is shown in Figure 3. It is made up of an O-ring and a base groove with the motor shell encapsulation. The shell in the axial direction (*Z*-axis) compresses the O-ring. Three bolts with a preload of 50 N each apply pressure on the whole structure. In order to reduce the number of mesh, the three bolts use beam elements instead of the bolt 3D structure model. The shell compresses the O-ring to the base groove, which achieves the seal function. There is a gap between the groove and O-ring in the radial direction as Figure 3 shows. When the shell compresses the O-ring, there should be an equation that the section area A (Figure 3b) is less than B (Figure 3c). This is to ensure that the O-ring’s maximum deformation material does not overflow the groove and affect the sealing performance and ensure that there is sufficient space for the O-ring to deform during compression. After the O-ring is compressed, there is remaining space in the groove. This can ensure that the O-ring material will not fail due to insufficient groove space. Figure 3d shows five contact surfaces, which include three contact surfaces of the O-ring. The contact surface 1 is the red color, the contact surface 2 is the blue color and the contact surface 3 is the green color. Contact surfaces 4 and 5 are the contacts between the motor base and shell. The contact pressure could prevent the water pressure, making it meet the requirements of waterproofing when the motor sealing structure works.

The motor shell material is metal, which is typically an isotropic linear material. Therefore, Hooke’s law describes stress–strain relationships.

(2) $$\left\{\begin{array}{c} \varepsilon_{x}=\frac{1}{E}\;\left[\sigma_{x}-\mu \left(\sigma_{y}+\sigma_{z}\right)\right]\\ \varepsilon_{y}=\frac{1}{E}\;\left[\sigma_{y}-\mu \left(\sigma_{x}+\sigma_{z}\right)\right]\\ \varepsilon_{z}=\frac{1}{E}\;\left[\sigma_{z}-\mu \left(\sigma_{x}+\sigma_{y}\right)\right] \end{array}\right.$$

where *μ* is the Poisson’s ratio, *E* is the elastic modulus, and $\varepsilon_{x}$, $\varepsilon_{y}$, and $\varepsilon_{z}$ are the strain components.

The von Mises stress (equivalent stress) is calculated based on the fourth strength theory (shape change specific energy theory). Its core idea is to map complex multiaxial stress states to uniaxial yield strength through an equivalent method for evaluation. Generally, higher magnitudes of von Mises stress generally correlate with an increased propensity for material failure [42,43,44]. The von Mises stress σ is defined as

(3) $$\sigma =\sqrt{\frac{1}{2}[{\left(\sigma_{1}-\sigma_{2}\right)}^{2}+{\left(\sigma_{2}-\sigma_{3}\right)}^{2}+{\left(\sigma_{3}-\sigma_{1}\right)}^{2}]}$$

where $\sigma_{1},\;\sigma_{2},and\;\sigma_{3\;}$ are the principal stresses in three directions of an element, respectively [41].

Many studies mention that since the hardness of metal materials is much greater than that of rubber materials, the metal materials are simplified into rigid bodies and then simulated and analyzed, which can reduce the amount of calculation. However, the simplified model is not suitable for models with complex geometries because the simplified model cannot reflect the details of the mechanical information. Therefore, a 3D model, which contains the structure details, is introduced for analysis in this study.

The finite element analysis model and method in this study are based on the methods of previous studies. The multi-objective optimization method for searching for the optimal design scheme is the focus of this study.

In this study, the groove depth varies from 0.6 to 1.1 mm. The preload of each bolt is 50 N, and the impact of varying groove depths on the sealing performance is studied. The material for the motor shell is S45C (structural steel), while the base and cover are made of ADC12 (aluminum alloy); the details of material properties are presented in Table 1.

There are a total of five contact surfaces in the model and three of them are around the O-ring, which is in Figure 3d. The friction coefficient of contact surfaces is listed in Table 2. The connection between the shell and cover is a bond connection. In the ANSYS mechanical 2023R2 software, the O-ring surface is set as the contact body, and the base and shell as the target bodies. The contact behavior is set as frictional type.

### 2.3. Grid Independence Test

**Figure 4:**
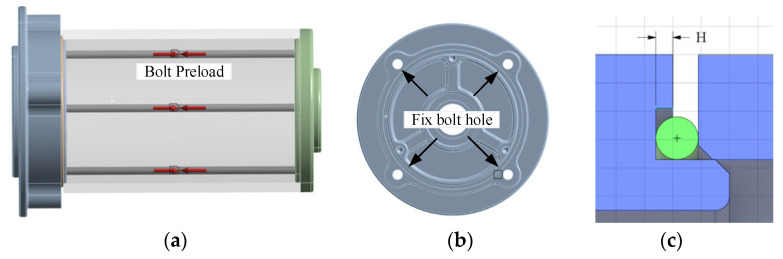
Boundary conditions: (**a**) preload position, (**b**) fixed constraint position, and (**c**) sealing groove.

Figure 4a shows bolt preload direction in encapsulation using beam element. The bolt preload presses the mating parts into each other, which creates contact pressure between them. Elements that transfer forces between the bolts and the assemblies are connections. Connections are special elements that are defined in the model. Ansys mechanical provides several different types of connections such as contact connections, joint connections, and beam connections. Using solid elements with frictional contacts in large assemblies with multiple bolts can make simulations computationally very expensive (calculation time and workstation hardware configuration). There is no need for bolt geometric meshing of the beam element, and the model size is significantly reduced. When using beam connections, the geometries for bolts, washers, or nuts are all ignored. The O-ring is put into the groove, the shell is assembled in the Z direction through the contact surface 4 with a friction coefficient which is 0 as the contact surface 4 is gap fit. The O-ring is compressed as the preload increases to 50 N in Figure 4. The bolt hole is fixed in Figure 4b. Before the simulation, the ‘Stabilization’ option and ‘Large Deflection’ option are considered. Figure 4c shows the different H from 0.6 mm to 1.1 mm.

**Figure 5:**
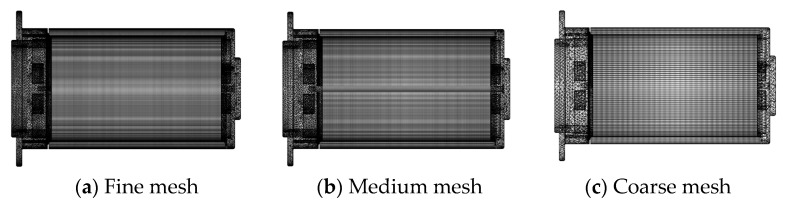
Grid independence test.

In order to minimize the influence of mesh on von Mises stress, contact pressure, O-ring deformation, and other items on the simulation results, the mesh independence of the finite element model with the same geometric model and the same boundary conditions was verified. This research meshes the model with the ANSYS mesh module. The coarse, medium, and fine mesh are shown in Figure 5, respectively.

Table 3 presents the simulated results. These simulation models consume 1128 min, 520 min, and 103 min. The table indicates that both the medium mesh model and fine mesh model simulation results are very close. The medium mesh model is adequate to solve the problem accurately. The fine mesh takes the longest time, which is 1128 min.

### 2.4. Optimization Method

**Figure 6:**
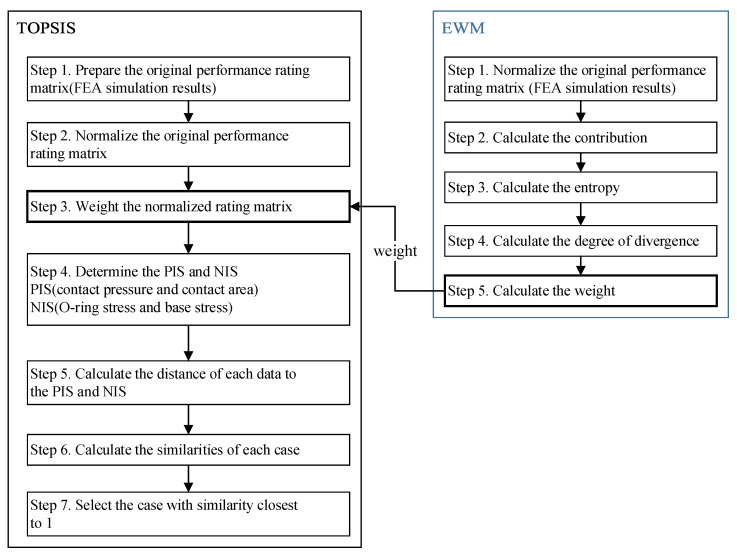
The flow chart of the E-TOPSIS method.

When the groove depth H changed, the simulation results changed in different trends. For the different design parameters, it is rather challenging to select the optimal design scheme solely through intuitive judgment. Experienced mechanical engineers have affirmed this parameter. The technique for order preference by similarity to ideal solution (TOPSIS) is a widely used method of multi-objective decision making to optimize parameters, which was proposed by Hwang and Yoon (1981) [45]. It encompasses both the ideal alternatives and the negative ideal alternatives and is convenient to calculate [46]. The entropy method is used to calculate the weight of each evaluation index [47]. The procedure of the optimization method is shown in Figure 6.

#### 2.4.1. The TOPSIS Method

The TOPSIS method calculates the similarity of each scheme according to the distance between each scheme and the PIS (positive ideal solution) and the NIS (negative ideal solution). The whole process can be summed up in seven key steps.

Step 1: Make the original performance matrix.

(4) $$\begin{array}{cc} & \begin{array}{ccccc} C_{1} & \;C_{2}\; & C_{3} & - & C_{n} \end{array}\\ \begin{array}{c} A_{1}\\ A_{2}\\ A_{3}\\ -\\ A_{m} \end{array} & \left[\begin{array}{ccccc} x_{11} & x_{12} & x_{13} & - & x_{1n}\\ x_{21} & x_{22} & x_{23} & - & x_{2n}\\ x_{31} & x_{32} & x_{33} & - & x_{3n}\\ - & - & - & - & -\\ x_{m1} & x_{m2} & x_{m3} & - & x_{mn} \end{array}\right] \end{array}$$

where *A_i_* (*i* = 1, 2, 3, …, *m*) is the *i*th value of H, and *C_j_* (*j* = 1, 2, 3, …, *n*) is the *j*th evaluation index (i.e., contact pressure, contact area, and stress).

Step 2: Normalize the matrix.

(5) $$r_{ij}=x_{ij}/\sqrt{\sum_{i=1}^{m}x_{ij}^{2}},i=1,2,3,\ldots ,m\;\mathrm{and}\;j=1,2,3,\ldots ,n$$

Step 3: Establish the weighted normalized decision matrix.

(6) $$v_{ij}=w_{j}r_{ij},i=1,2,3,\ldots ,m\;\mathrm{and}\;j=1,2,3,\ldots ,n$$

where *w_j_* is the weight for index *C_j_*.

Step 4: Choose PIS (contact pressure and area) and NIS (stress).

(7) $$PIS=\left\{\left(\underset{i}{\min}v_{ij}|j\in J_{1}\right),\left(\underset{i}{\max}v_{ij}|j\in J_{2}\right),|i=1,2,3,\ldots ,m\right\}$$

(8) $$NIS=\left\{\left(\underset{i}{\max}v_{ij}|j\in J_{1}\right),\left(\underset{i}{\min}v_{ij}|j\in J_{2}\right),|i=1,2,3,\ldots ,m\right\}$$

where *J*_1_ and *J*_2_ are the cost and the benefit attributes, respectively.

Step 5: Compute each alternative distance to the PIS and the NIS.

The following formula calculates the distance to the PIS, where $v_{j}^{+}$ is the value of the *j*th element of PIS.

(9) $$D_{i}^{+}=\sqrt{\sum_{j=1}^{n}{\left[v_{ij}-v_{j}^{+}\right]}^{2}},\;i=1,2,3,\ldots ,m$$

The following formula calculates the distance to the NIS, where $v_{j}^{-}$ is the value of the *j*th element of NIS.

(10) $$D_{i}^{-}=\sqrt{\sum_{j=1}^{n}{\left[v_{ij}-v_{j}^{-}\right]}^{2}},\;i=1,2,3,\ldots ,m$$

Step 6: Obtain the similarity of each alternative to the ideal solution.

(11) $$C_{i}^{*}=\frac{D_{i}^{-}}{D_{i}^{+}+D_{i}^{-}},\;i=1,2,3,\ldots ,m$$

where C_i_^*^ ∈ [0,1] ∀*i* = 1, 2, 3, …, m.

Step 7: C_i_^*^ closest to 1 is the optimal solution.

#### 2.4.2. Entropy Weight Method

The weight of the simulation results is calculated by the entropy method, which is based on Shannons’s entropy theory [47]. This process is summed up in five key steps.

Step 1: Normalize the rating matrix [48].

For benefit physical quantity:

(12) $$x_{ij}\prime =\frac{x_{ij}-x_{j}^{min}}{x_{j}^{max}-x_{j}^{min}},i=1,2,3,\ldots ,m$$

For cost-type physical quantity:

(13) $$x_{ij}\prime =\frac{x_{j}^{max}-x_{ij}}{x_{j}^{max}-x_{j}^{min}},i=1,2,3,\ldots ,m$$

Step 2: Obtain the contribution of the *i*th alternative in the *j*th index.

(14) $$p_{ij}=x_{ij}\prime /\sum_{i=1}^{m}x_{ij}\prime ,i=1,2,3,\ldots ,m\;\mathrm{and}\;j=1,2,3,\ldots ,n$$

Step 3: Compute the entropy for each index.

(15) $$E_{j}=-K\sum_{i=1}^{m}p_{ij}\ln p_{ij},\;K=1/\ln m,j=1,2,3,\ldots ,n$$

where 0 ≤ *E_j_* ≤ 1.

Step 4: Compute the degree of divergence.

(16) $$d_{j}=1-E_{j}$$

Step 5: Compute the weight.

(17) $$w_{j}=d_{j}/\sum_{k=1}^{n}d_{k}$$

## 3. Sealing Performance Analysis

### 3.1. FEM Method Analysis

#### 3.1.1. Metal Structure Analysis

**Figure 7:**
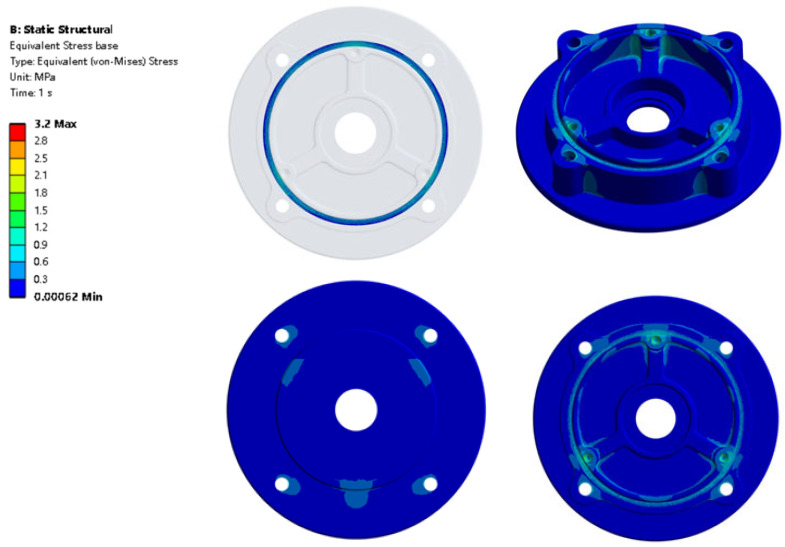
Motor base von Mises stress with groove depth H = 0.6 mm.

**Figure 8:**
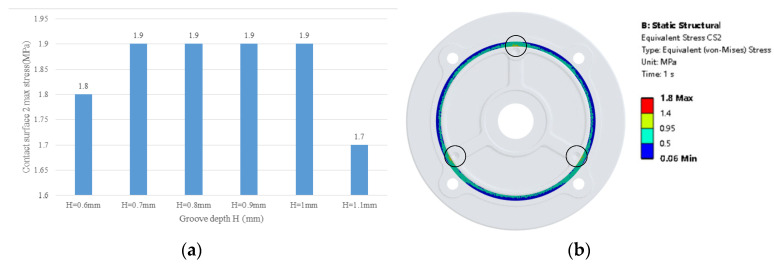
Motor base contact surface 2 von Mises stress distribution: (**a**) contact surface 2 max von Mises stress with different groove depth; (**b**) contact surface 2 von Mises stress distribution of H = 0.6 mm.

Figure 7 shows the motor base’s stress distribution of the simulation result with the maximum von Mises stress in the bolt hole. However, the von Mises stress in the contact surface 2 is not uniformly distributed along the perimeter direction, which is different from other studies. The location of the stress around the bolt hole is greater than that in the other surface. The maximum von Mises stress is 3.2 MPa, which is less than the aluminum alloy tensile yield stress which is 280 MPa. From Figure 8, when the groove depth H = 1.1 mm, the max stress is 1.7 MPa which is the lowest among all the cases. The stress concentration zones are in the black circle in Figure 8. The stress distribution is almost the same through all the cases from H = 0.6 to H = 1.1 mm. The stress is larger at the bolt hole while smaller at the position between the two bolt holes. The stress distribution is not uniform in the circumference direction. Large stress can affect the stability of the motor base structure, and thus affect the seal durability.

#### 3.1.2. O-Ring Simulation Result

**Figure 9:**
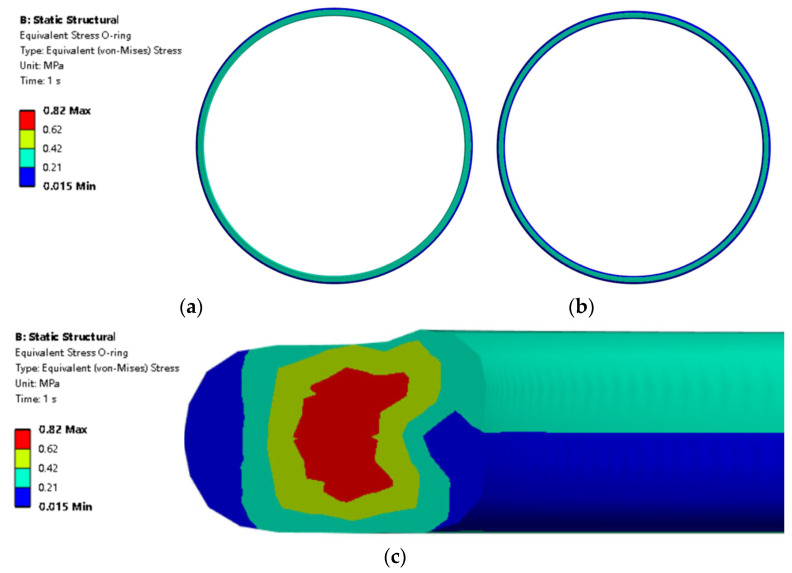
O-ring von Mises stress (**a**) stress at CS1 side and (**b**) stress at CS2 side, and (**c**) O-ring section stress distribution.

The O-ring’s material is NBR, and the material property is that tensile yield stress is 5.292 MPa and the tensile ultimate stress is 33 MPa. The mesh density of the O-ring is important and the medium mesh is used in this study. Larger mesh sizes are easier and lead to calculation errors, while smaller mesh sizes may cause the occurrence of stress singularities. Inappropriate mesh sizes can also result in mesh distortion during the calculation process, thereby causing calculation errors. The stress distribution of the O-ring when H = 0.6 mm is shown in Figure 9. The O-ring surface stress distribution on the contact surface 1 side is larger than that on the surface 2 side from Figure 9a,b. Figure 9c shows that the max stress is 0.82 MPa, which is lower than the yield stress of 5.929 MPa. The O-ring section reveals that at the chamfered area, there exist several minor protrusions on the interior surface of the O-ring.

**Figure 10:**
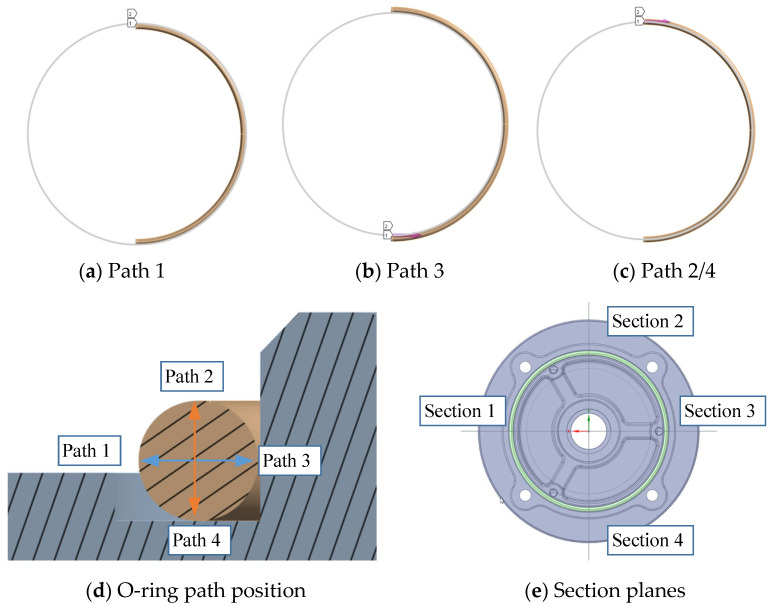
O-ring construction geometry.

The rubber is compressed when the shell package is on the base. There are 4 path lines in the O-ring surface to evaluate the deformation of the rubber seal in the circumferential direction and the axial direction. Path 1 is shown in Figure 10a. Path 3 is shown in Figure 10b. Two planes perpendicular to each other with the O-ring intersect into four section planes; the four section planes establish four circle lines that describe the O-ring deformation from the circle to flat shape. The two planes perpendicular to each other are shown in Figure 10e. One of the section planes of the O-ring is shown in Figure 10d.

**Figure 11:**
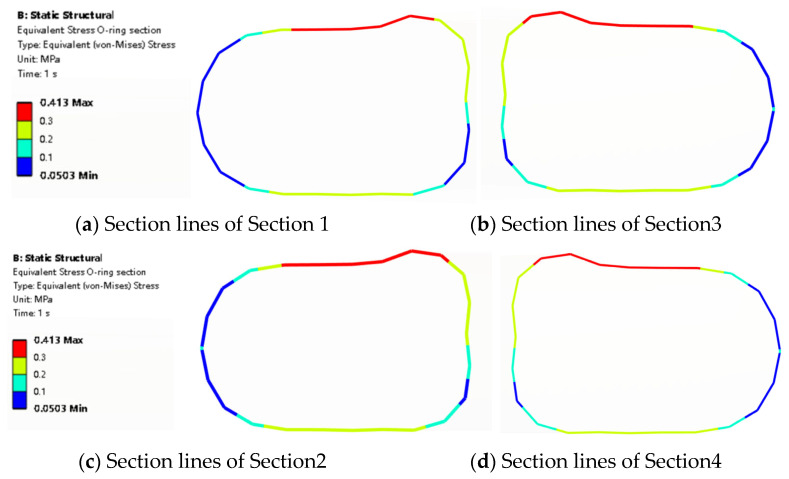
O-ring section line stress distribution.

In Figure 11, the section lines are formed by the intersection of two surfaces which are the section planes in Figure 10e and the O-ring surface. The von Mises stress results show that when the O-ring is compressed, the surface stress is asymmetric, and the stress is larger at the contact surface 1 side than contact surface 2 side in Figure 11. This indicates that contact surface 1 is more prone to damage. During the virtual design phase, the stress of contact surface 1 should be considered first to ensure the reliability of the sealing structure. Furthermore, design reinforcement at the CS1 region also needs to be considered. The stress distribution is different among the four section lines. Section 2 and Section 4 are the same while they are at the same distance from the bolt hole.

**Figure 12:**
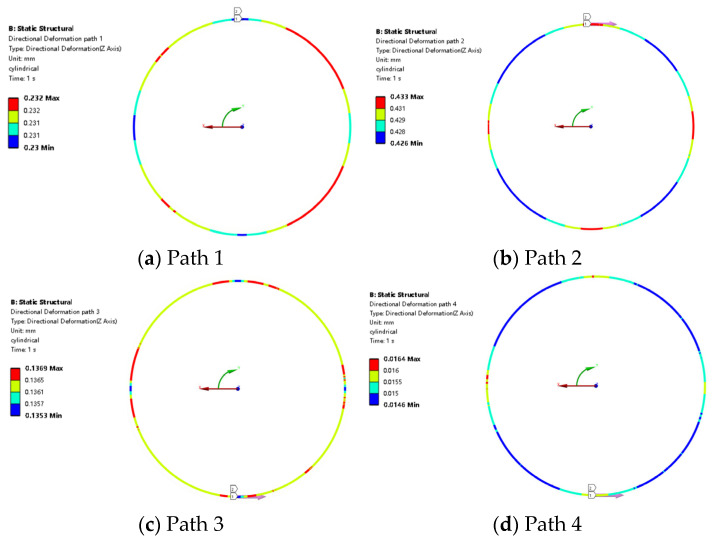
O-ring path deformation at Z direction.

The cylindrical coordinate system is established to study the circumferential and axial deformation and stress of the O-ring along the path from path 1 to path 4. The cylindrical coordinate system has the same X direction and Z direction as the Global Coordinate System, which is shown in Figure 12. The results show that the O-ring deformation in the X direction is different. That means the deformation is different in different areas.

At the same direction (Z direction), the max deformation position is not the same which is shown in the red color in Figure 12. According to Figure 12a, the path 1 deformation in the Z direction shows that the deformation fluctuates along the path. According to Figure 12b, deformation in the Z direction shows that the deformation is regular. According to Figure 12c, the deformation becomes random fluctuation along path 3. This indicates that there will be greater randomness in the sealing performance of contact surface 3 during the sealing performance. Path 4 deformation is the smallest among all the paths, which Figure 12d shows. Although the value of the change is small, the above results show that the deformation of the rubber ring is inconsistent in the circumference.

**Figure 13:**
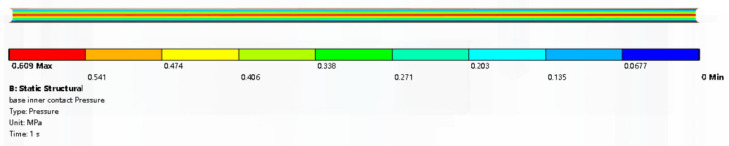
Contact pressure of CS3.

**Figure 14:**
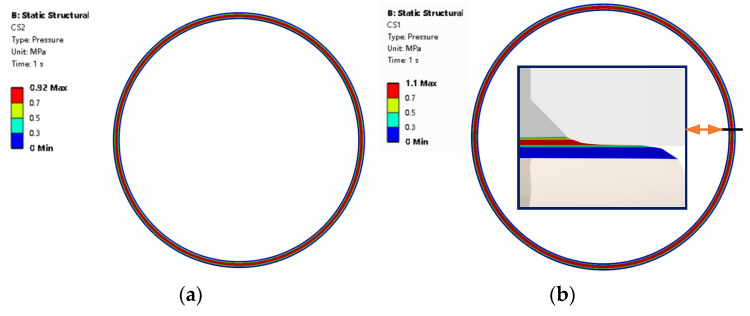
Contact pressure (**a**) contact pressure of contact surface 2, (**b**) contact pressure of contact surface 1 and its cross-section.

The largest contact pressure is located in the middle of the O-ring surface around path 2 and path 4. While contact pressure is smaller on the surface around paths 1 and 3. The max contact pressure of CS3 is 0.609 MPa at the position around path 3, which is shown in Figure 13. The max contact pressure of CS2 is 0.92 MPa at the position around path 4 shown in Figure 14a. The max contact pressure of CS1 is 1.1 MPa at the position around path 2 shown in Figure 14b. The contact pressure of CS1 is different from that CS2 and CS3. The max contact pressure is in the corner of the chamfer on the shell. The rubber seal sticks firmly to the top chamfer and the contact pressure is in the range from 0.7 MPa to 1.1 MPa.

### 3.2. Experimental Verification

#### 3.2.1. Friction Coefficient Test

The coefficient of friction (COF) is a basic concept in the field of tribology. It quantifies the resistance of motion between two contact surfaces and measures the difficulty of sliding one object over another. It is calculated by finding the average tension during the test and dividing it by the weight of the object. For further study of the friction coefficient of rubber seal and metal, the experimental test was taken into place. The μk (friction coefficient) formula is

(18) $$\mu k=\frac{F}{N}$$

where N is the normal force while F is the frictional force.

Before conducting the experiment, the surface of the subject was clean to ensure that there was no debris, which would affect the experiment. The surface roughness of the friction test object was the same as the surface roughness of the components used in the waterproof test sample.

The normal force N was 2.21 kg and the O-ring was dragged at a constant speed to achieve the F that equals frictional force. Each experiment was repeated five times in the same way and the test data is listed in Table 4. By analyzing the data in Table 4, it can be concluded that μk is 0.17 ± 0.01. The average value of the friction coefficient is 0.17; the standard deviation of the friction coefficient is 0.01.

The friction coefficient between aluminum alloy and structure steel was also tested in this study. Each experiment was repeated five times in the same way. The test data is in Table 5. By analyzing the data in Table 5, it can be concluded that μk is 0.15 ± 0.01. The average value of the friction coefficient is 0.15; the standard deviation of the friction coefficient is 0.01.

The friction coefficient between aluminum alloy materials with rubber was also tested in this study. Each experiment was repeated five times in the same way. Aluminum alloy with rubber frictional coefficient test data is listed in Table 6. By analyzing the data in Table 6, it can be concluded that μk is 0.18 ± 0.01. The average value of the friction coefficient is 0.18; the standard deviation of the friction coefficient is 0.01.

#### 3.2.2. Waterproof Performance Test

According to standards IEC 60529 and ISO 20653:2013, for the IPX7 experiment, the motor shall be immersed in water to a depth of up to one meter for a minimum duration of 30 min.

**Figure 15:**
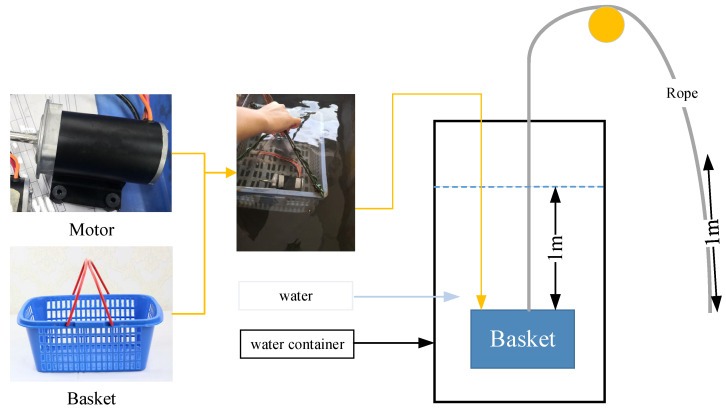
IPX7 experimental process.

In order to test the waterproof performance, a real motor was manufactured according to the size obtained from the multi-objective optimization algorithm results shown in Figure 15. The sealing performance of the O-ring is evaluated by sealing all the other connections with adhesive, isolating the O-ring as the sole sealing component, and conducting the test in a static water tank. The motor was put into the prepared basket, and then the basket was lifted with a rope. The basket was slowly immerseed with the motor into the water to a depth of 1 m through the rope. After immersing for 30 min, the basket was pulled out of the water. The experiment was performed under stable ambient conditions at 25 °C, with no water turbulence or pressure fluctuations observed during the testing process. The water pressure is calculated with the formula below:

(19) P=ρgh

where P is pressure in the water, ρ is the density of water, g is the acceleration of gravity, and h is the depth immersed in water.

So, for IPX7, $\rho =1000\;kg/m^{3}$, g = 10 m/s^2^, h = 1 m, the water pressure is 0.01 MPa.

## 4. Results and Discussion

The von Mises stress (O-ring and motor base) and the contact pressure and contact area are important physical quantities to evaluate the sealing performance. For the sealing structure, the contact pressure should be larger than the water pressure that IPX7 requires. The von Mises stress needs to be less than the yield stress of the material.

### 4.1. Influence of Groove Depth Parameters

**Figure 16:**
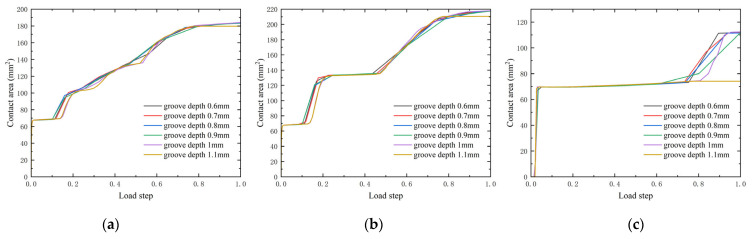
Contact area with different groove depth (**a**) contact area of CS1 with different groove depth, (**b**) contact area of CS2 with different groove depth, (**c**) contact area of CS3 with different groove depth.

On the premise of good element quality (0.88) of the finite element model, we calculated the finite element model and analyzed the calculation results. The contact area is different when the groove depth is changed from 0.6 to 1.1 mm. As the preload increases to 50 N, the contact area increases. Figure 16 plots the change in contact surface area with bolt preload increase to 50 N. Divide the entire loading process into 10 load steps. For example, 0.2 on the *X*-axis in the figure represents the second load step. As the load increases, the contact area is increased which may fill the groove. The relationship between the contact area and the sealing performance is the more the contact area, the better the sealing performance [49]. Figure 16 indicates that when the O-ring works, the main waterproof function is the contact surface 1 and 2 as the contact surface 3’s contact area is about half of that of the other two contact surfaces. However, contact surface 3 also helps provide another contact surface, which may contribute to axial stabilization and help improve sealing performance. When the groove depth is 0.6 to 0.9 mm, the contact area may increase earlier than 1–1.1 mm, so may the contact surface 2.

Contact surfaces 1 and 2 have the same tendency, which is at the first load step the contact area remains almost the same while it suddenly rises after the first load step. When the load step increases to the eighth load step (at the position of 0.8 on the *x*-axis in Figure 16) the contact area remains the same. The 10 load steps could summarize three main stages, which may affect the contact area. That is the first stage when the preload increases to 5 N, the second stage when the preload increases to 40 N, and the third stage when the preload is complete to 50 N.

For the contact surface 2 at the second stage, there may be another two stages which are from 10 N to 25 N, and the contact area fluctuates constantly. This is different from the contact surface 3, which keeps the contacting area almost constant until the eighth load step. Contact surface 3 has the same trend as contact surface 1 and contact surface 2 during stage two. When the groove depth H = 1.1 mm, the contact surface area remains the same from stage two to stage three, in which the contact area is the smallest in all the contact surface areas.

The contact surface 2 area is more than 200 mm^2^, the contact surface 1 area is about 180 mm^2^, and the contact surface 3 area is less than 120 mm^2^. It indicates that the contact surface 2 has the largest contact area.

### 4.2. Optimal Design by E-TOPSIS Method

The simulation results are contact surface 1–3’s contact area, contact surface 1–3’s contact pressure, O-ring stress, and base stress under various groove depths. These parameters directly affect the sealing performance and the stability of the motor structure. The value of these indices changes in different trends regarding groove depths. Therefore, a multi-criteria performance optimization method needs to select the optimal groove depth.

For the cases satisfying the requirement of GB T3452.3-2005, the parameters for the entropy method and TOPSIS method are listed in Table 7. The similarity that is closest to 1 represents the optimal design. There are eight physical quantities from the simulation result that are considered to optimize the groove depth. The positive ideal solution pertains to the contact area and contact pressure that are conducive to waterproofing, while the negative ideal solution refers to the component von Mises stress, which may have an impact on the structural stability.

**Figure 17:**
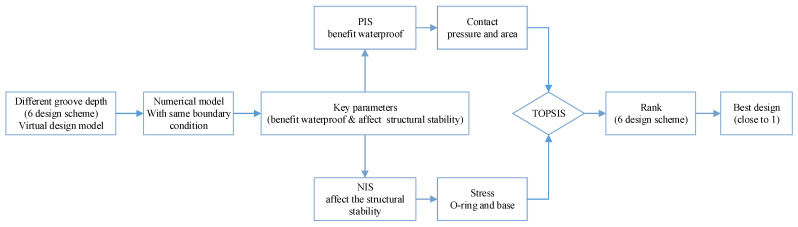
The process of finding the optimal design scheme by the TOPSIS method.

The positive ideal solution represents the optimal solution (scheme) under a given assumption, where its attribute values attain the best values among all the alternatives. In contrast, the negative ideal solution denotes the worst solution (scheme) under the same assumption, with its attribute values reaching the worst values among all the alternatives. The rule of scheme ranking is to compare the alternatives with the positive ideal solution and the negative ideal solution. If one of them is closest to the positive ideal solution, but at the same time far away from the negative ideal solution, then the scheme is the best one among the alternatives (in this study it is the six different groove depth design schemes that are list in Table 7). The whole process of finding the optimal design scheme by the TOPSIS method is shown in Figure 17.

The similarities calculated by the TOPSIS method under various groove depths are presented in Table 8. According to the similarities and corresponding ranks, the optimal design is a groove depth of 0.9 mm. The similarity for H = 1 mm is very close to that for H = 0.9 mm. The contact pressure and contact area of H = 1.1 mm are the smallest among all the design schemes. The design scheme with a groove depth of 1.1 mm scored the lowest among all the schemes, and it is the worst design scheme.

### 4.3. Waterproof Experimental Verification

**Figure 18:**
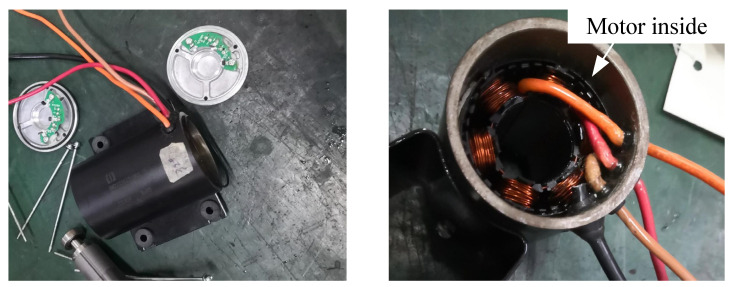
Waterproof experimental verification.

We manufactured a prototype with a groove depth is 0.9 mm, which is presented in Table 8. We sealed the other gap structures of motors except for the O-ring. The test conditions were according to the test method of IEC60529. One prototype was tested with a temperature range of 15–35 °C, relative humidity of 25–75%, and air pressure of 0.086–0.106 MPa. This experiment can be reproduced through the test methods and environmental conditions described in this paper. We put the motor into the basket, hoisted the basket with a soft rope, and slowly sank the basket into the water. We controlled the depth of the basket immersed in the water by the length of the rope. At this time, the length of the rope immersed in the water should be one meter. We kept the basket stationary at a water depth of one meter for 30 min. Then, we hoisted the basket out of the water through the rope. As Figure 18 shows, in the process of diving underwater of the test motor, the test motor is placed in a basket overhang by a rope while the depth of the motor is controlled by the length of the rope. After the test, the motor was disassembled to check the sealing performance of the rubber sealing structure. We removed the water stains on the surface of the motor. There was no water appearing as Figure 18 shows inside the motor.

## 5. Conclusions

This study aims to apply the E-TOPSIS method to optimize multi-parameter evaluation on motor seal structure. In addition, under the condition of the IPX7 test standard, conduct experimental verification on the optimized model to verify the reliability of the O-ring sealing performance. As a comparison, the corresponding impacts of the reference physical quantity are also studied. Detailed conclusions as below.

(1) The deformation distribution and contact pressure distribution of the rubber seal calculated in the 3D model are not exactly the same along the circumferential direction, which is different from the equal distribution mentioned in many papers [21,25,50,51,52]; thus, simplifying the model into a two-dimensional model is not a very accurate simulation in a certain condition, especially to those structures which are not consistent along the circumference direction. It is not recommended to use the two-dimensional simplification method for asymmetric geometric shapes. The O-ring stress of contact surface 1 side is larger than that of contact surface 2 side, which means materials in this area are more likely to be damaged than in other areas.
(2) In this study, the technique for order preference by similarity to the ideal solution (TOPSIS) method is introduced to order the optimal solutions. It is concluded that when the diameter of the O-ring is 1.5 mm, the optimal groove depth is 0.9 mm. The proportional relationship between the diameter D of the O-ring and the optimal groove depth can be extended to H = 0.6 D. This method of analyzing the influence of parameter changes on the O-ring seal performance can reduce test time and improve the economy and timeliness of the O-ring seal design.
(3) Through the experimental verification, it is proved that the optimization algorithm selects the optimal design scheme that sealing performance in this study meets the requirements of IPX7. There was no water inside the motor. No water leakage occurred after its disassembly.
(4) From the perspective of application scenarios, the method proposed in this paper is not only suitable for the rubber sealing performance optimization of motors but also applicable to optimization in other fields. Wherever many tasks with similarities can be constructed in optimization, this method can be effectively applied.

The proposed method is well suited for complex industrial products with many evaluation parameters, particularly when AI-based extraction is integrated. This method is very beneficial for the handling of complex structures or problems in industrial products. Often, the evaluations of these issues are so complex that it is difficult to assess the merits in all respects in different trends. From this perspective, this paper provides a new application optimization paradigm combining data and mechanisms in the field of industrial product optimization, offering a broad research prospect.

Furthermore, there are several limitations in this study.

(1) The finite element model takes into account the nonlinear characteristics of the material, which results in a computation time of up to nine hours for a single model. In this study, six models were compared, and the long computation time became a limitation. Due to the extended calculation time, the number of models that could be evaluated was restricted. For comparing more design options in the future, accelerating the simulation speed will be a key consideration.
(2) This paper only evaluates the comparison results between different groove depths under the same bolt preload and uses the E-TOPSIS method to optimize these results. It does not compare the combined design schemes of different bolt preloads and different groove depths.
(3) This study applies the E-TOPSIS algorithm for multi-objective optimization. With the development and application of artificial intelligence technology, the combination of artificial intelligence algorithms and multi-objective optimization algorithms, when applied to the design of sealing structures, will be a key research focus in the future and will have broad prospects.

## Figures and Tables

**Table 1:** Material properties of motor.

Material	E (MPa)	*μ*	R_p0.2_ (MPa)	*R_m_* (MPa)
ADC12	71,000	0.33	280	310
S45C	200,000	0.3	899	1029

**Table 2:** Friction coefficient of contact surface.

Contact Surface	1	2	3	4	5
Friction coefficient	0.18	0.18	0.18	0	0.15

**Table 3:** Grid independence test.

	Fine	Medium	Coarse
Mesh elements	610,140	368,710	166,300
O-ring max stress (MPa)	0.824	0.818	0.807
Total deformation (mm)	0.435	0.433	0.431
CS1 CP (MPa)	1.109	1.104	1.101
CS2 CP (MPa)	1.018	0.927	0.918
Time to solve	18 h 48 m 3 s	8 h 40 m 28 s	1 h 43 m 55 s

**Table 4:** Rubber with steel frictional coefficient test data.

Test	N (N)	F (N)	μk
1	22.10	4.00	0.18
2	22.10	3.80	0.17
3	22.10	3.90	0.18
4	22.10	3.40	0.15
5	22.10	4.00	0.18

**Table 5:** Aluminum alloy and structure steel frictional coefficient test data.

Test	N (N)	F (N)	μk
1	26.65	4.20	0.16
2	26.65	4.00	0.15
3	26.65	4.00	0.15
4	26.65	4.20	0.16
5	26.65	4.00	0.15

**Table 6:** Aluminum alloy with rubber frictional coefficient test data.

Test	N (N)	F (N)	μk
1	22.10	3.80	0.17
2	22.10	4.20	0.19
3	22.10	4.00	0.18
4	22.10	3.60	0.16
5	22.10	4.00	0.18

**Table 7:** The parameters for the optimization method.

Groove Depth (mm)	0.6	0.7	0.8	0.9	1	1.1
Contact pressure CS1 (MPa)	1.121	1.123	1.110	1.122	1.124	0.982
Contact pressure CS2 (MPa)	0.931	0.926	0.930	0.930	0.928	0.800
Contact pressure CS3 (MPa)	0.622	0.607	0.618	0.607	0.605	0.487
Contact area CS1 (mm^2^)	183.628	183.312	183.217	183.514	184.280	179.780
Contact area CS2 (mm^2^)	217.368	217.377	217.341	217.474	217.900	210.630
Contact area CS3 (mm^2^)	111.857	111.864	111.499	111.889	112.440	74.140
Stress O-ring (MPa)	0.830	0.829	0.829	0.831	0.808	0.691
Stress Base (MPa)	3.250	3.788	3.983	2.842	3.137	2.655

**Table 8:** Similarities and rankings of different groove depths.

Groove Depth (mm)	0.6	0.7	0.8	0.9	1	1.1
Pi	0.904	0.859	0.842	0.926	0.919	0.154
Rank	3	4	5	1	2	6

## Data Availability

The original contributions presented in this study are included in the article. Further inquiries can be directed to the corresponding author.

## Nomenclature

Symbol	Unit	Implication
*σ*	[MPa]	von Mises stress
σ_1_	[MPa]	principal stresses at x direction
σ_2_	[MPa]	principal stresses at y direction
σ_3_	[MPa]	principal stresses at z direction
*E*	[MPa]	Young’s modulus
*R_p_*_0.2_	[MPa]	Tensile Yield Strength
*R_m_*	[MPa]	Tensile Ultimate Strength
*N*	[N]	applied normal force
*F*	[N]	frictional force
*P*	[MPa]	water pressure
g	[m/s^2^]	Earth gravity
h	[m]	submersion in water depths
*H*	[mm]	groove depth
*x*, *y*, *z*	[mm]	Cartesian coordinates
*ρ*	[kg/m^3^]	water density

## Abbreviations

Abbreviation	Full Name
IP	Ingress Protection
NIS	negative ideal solution
PIS	positive ideal solution
TOPSIS	technique for order preference by similarity to the ideal solution
*NBR*	nitrile butadiene rubber
*μ*	Poisson’s Ratio
*CS1*	contact surface 1
*CS2*	contact surface 2
*CS3*	contact surface 3
*CS4*	contact surface 4
*CS5*	contact surface 5
*μk*	kinetic frictional coefficient
*COF*	coefficient of friction
FEM	Finite Element Method
EWM	Entropy Weight Method
